# Ginkgolic acid improves bleomycin-induced pulmonary fibrosis by inhibiting SMAD4 SUMOylation

**DOI:** 10.1155/2022/8002566

**Published:** 2022-06-06

**Authors:** Lan Yu, Xiyun Bian, Chunyan Zhang, Zhouying Wu, Na Huang, Jie Yang, Wen Jin, Zongqi Feng, Dongfang Li, Xue Huo, Ting Wu, Zhongmin Jiang, Xiaozhi Liu, Dejun Sun

**Affiliations:** ^1^Clinical Medical Research Center/ Inner Mongolia Key Laboratory of Gene Regulation of the Metabolic Diseases, Inner Mongolia People's Hospital, Hohhot 010017, China; ^2^Department of Endocrine and Metabolic Diseases, Inner Mongolia People's Hospital, , Hohhot 010017 , China; ^3^Department of Central Laboratory, The Fifth Central Hospital of Tianjin, Tianjin 300450, China; ^4^Tianjin Key Laboratory of Epigenetics for Organ Development in Preterm Infants, The Fifth Central Hospital of Tianjin, Tianjin 300450, China; ^5^Department of Pathology, The Fifth Central Hospital of Tianjin, Tianjin 300450, China; ^6^Department of Pulmonary and Critical Care Medicine/ Key Laboratory of National Health Commission for the Diagnosis & Treatment of COPD, Inner Mongolia People's Hospital, Hohhot, Inner Mongolia, China

## Abstract

Idiopathic pulmonary fibrosis (IPF) is a refractory chronic respiratory disease with progressively exacerbating symptoms and a high mortality rate. There are currently only two effective drugs for IPF; thus, there is an urgent need to develop new therapeutics. Previous experiments have shown that ginkgolic acid (GA), as a SUMO-1 inhibitor, exerted an inhibitory effect on cardiac fibrosis induced by myocardial infarction. Regarding the pathogenesis of PF, previous studies have concluded that small ubiquitin-like modifier (SUMO) polypeptides bind multiple target proteins and participate in fibrosis of multiple organs, including PF. In this study, we found altered expression of SUMO family members in lung tissues from IPF patients. GA mediated the reduced expression of SUMO1/2/3 and the overexpression of SENP1 in a PF mouse model, which improved PF phenotypes. At the same time, the protective effect of GA on PF was also confirmed in the SENP1-KO transgenic mice model. Subsequent experiments showed that SUMOylation of SMAD4 was involved in PF. It was inhibited by TGF-*β*1, but GA could reverse the effects of TGF-*β*1. SENP1 also inhibited the SUMOylation of SMAD4 and then participated in epithelial-mesenchymal transition (EMT) downstream of TGF-*β*1. We also found that SENP1 regulation of SMAD4 SUMOylation affected reactive oxygen species (ROS) production during TGF-*β*1-induced EMT and that GA prevented this oxidative stress through SENP1. Therefore, GA may inhibit the SUMOylation of SMAD4 through SENP1 and participate in TGF-*β*1-mediated pulmonary EMT, all of which reduce the degree of PF. This study provided potential novel targets and a new alternative for the future clinical testing in PF.

## 1. Introduction

Idiopathic pulmonary fibrosis (IPF) is a chronic, progressive, and often fatal interstitial lung disease [1, 2]. Currently, the etiology and underlying mechanisms of IPF are unclear. It is generally believed that IPF lesions are caused by the combined action of inflammation, immune responses, lung injury, and fibrosis [3]. Pirfenidone and nintedanib are currently the only two approved oral preparations for IPF treatment [4–6]. However, the therapeutic efficacy of these two drugs is limited, and they have obvious gastrointestinal side effects [6, 7]. Therefore, there is an urgent clinical need to develop new more effective drugs for IPF treatment. Ginkgolic acid (GA) is a urushiol that primarily exists in the epicarp of *Ginkgo biloba* [8]. GA has a wide range of pharmacological effects, including antitumor [9], antioxidative [10], antiinflammatory [11], and antiviral [12]. Additionally, GA has an inhibitory effect on the myocardial fibrosis caused by cardiac infarction [13]. However, little is known about the effect of GA on IPF and its mechanism, which urgently needs to be further studied.

Small ubiquitin-like modifier (SUMO) is a small polypeptide similar to ubiquitin in structure but different in function [14]. Currently, there are at least five SUMO subtypes, namely, SUMO1–5 [15]. SUMOylation is a dynamic and reversible process [16]. Under the action of E1 activating enzymes (SAE1, SAE2), an E2 binding enzyme (Ubc9), and E3 ligases (PIAS1-3), SUMO molecules can be covalently attached to a substrate protein. Then, the covalently bound complex can undergo de-SUMOylation by the action of sentrin/SUMO-specific proteases (SENPs 1–3, and 5–7), and the peptide reenters a new SUMOylation pathway [17]. As an important posttranslational protein modification, SUMOylation has a wide range of regulatory effects on protein functions, including regulation of protein-protein interactions, transcriptional activity, DNA damage repair, and protein structural stability [18]. Recently, the role of SUMOylation in the lungs has been gradually uncovered, and it has been found that SUMOylation is closely related to lung diseases according to the target proteins [19, 20]. Additionally, SUMO1/SENP1 can be used as prognostic indicators for lung cancer survival [21], and inhibition of the SENP/HIF-1*α* pathway can alleviate acute lung injury (ALI) caused by lipopolysaccharide (LPS) [22]; the SUMOylation of HIF-1*α* by SUMO1 leads to its increased stability, which is an important mechanism for pulmonary vascular remodeling and pulmonary hypertension in chronic obstructive pulmonary disease (COPD) patients [23]; SUMOylation also plays an important role in *Mycoplasma pneumoniae*-induced pneumonia [24]. However, whether SUMOylation is involved in the process of pulmonary fibrosis (PF) remains unclear. Recently, it has been reported that GA can improve the cardiac fibrosis caused by myocardial infarction by inhibiting SUMO1 expression [13], which suggests its mechanism of action could be important in IPF.

TGF-*β*1 is the most important fibrogenic factor [25]. Previous studies have confirmed that TGF-*β*1 can induce lung fibroblasts to proliferate and transform into myofibroblasts, which ultimately cause lung fibrosis [26]. The SMAD family members are cytoplasmic signal transduction molecules involved in the TGF-*β* pathway that directly transfer TGF-*β* signals from the cell membrane to the nucleus [27] and participate in the regulation of a variety of cell activities, including cell proliferation, differentiation, migration, apoptosis, and maintaining internal homeostasis [28, 29]. There are eight members of the SMAD family, which are divided into three categories according to their structure and function: receptor-activated types (SMAD1, 2, 3, 5, and 8), the universal/adaptor type (SMAD4), and inhibitory types (SMAD6 and 7) [30]. Different SMADs mediate signals from different members of the TGF-*β* family [31]. However, it has been found that SMAD2 and SMAD3 are involved in TGF-*β* signal transduction in a rat model of IPF [32], while SMAD6 and SMAD7 inhibited this TGF-*β* activity [33]. Additionally, recent studies have shown that SMAD4 participates in PF through its nuclear localization and by promoting epithelial-mesenchymal transition (EMT) [32, 34]. It is not known whether SMAD4 SUMOylation is involved in IPF, and whether it is related to the protective effect of GA on PF. Herein, we first investigated the role of SUMOylation during IPF by evaluating clinical samples. Then, the therapeutic efficacy of GA on PF was tested *in vivo* and *in vitro*. Finally, we analyzed the molecular mechanism through which GA treatment improved IPF by investigating the SUMOylation of SMAD4. This study provides a new target and drug for IPF treatment, which could have a possible clinical significance.

## 2. Materials and Methods

### 2.1. Cell Culture and Treatment

Adenocarcinomic human alveolar basal epithelial cells A549 cells were obtained from ATCC (Manassas, VA, USA). Cells in logarithmic growth phase were digested with trypsin (Gibco/Thermo Fisher Scientific, Waltham, MA, USA) and resuspended in Roswell Park Memorial Institute (RPMI) 1640 medium containing 10% FBS, 100 U/mL penicillin, and 100 *μ*g/mL streptomycin (all from Gibco), and then cultured in a cell incubator with 5% CO_2_ at 37 °C. After the cells had adhered to the flask, they were treated with bleomycin (BLM; YZ-1076308), GA (SG8960, both from Solarbio Science & Technology, Beijing, China), or the two combined.

### 2.2. Plasmid Transfection

The overexpression plasmids pcDNA3.1-FLAG-SAMD4 and pcDNA3.1-HA-TGF-*β*1 were synthesized by Gemma Gene Company (Shanghai, China), and the pPLK/GFP + Puro-SENP1 shRNA lentiviral plasmid was purchased from the Public Protein/Plasmid Library (PPL01084-3a). A549 cells in logarithmic growth phase were transfected with the overexpression plasmids using Lipofectamine™ 2000 transfection reagent (Thermo Fisher Scientific); A549 cells stably expressing SENP1-shRNA were obtained after the lentiviral plasmid was transduced and positive cells were screened with puromycin.

### 2.3. Hematoxylin and Eosin (HE) Staining and Immunohistochemistry (IHC)

Clinical specimens were collected with the informed consent of the patient's family, and this study was approved by the Medical Ethics Committee of Tianjin Fifth Central Hospital (TJWZXYXEC-ky202015, 2020.07.27). Specimens, which fulfilled multidisciplinary diagnostic criteria described in the Official ATS/ERS/JRS/ALAT Clinical Practice Guideline, were collected from patients with IPF at the time of diagnostic surgical lung biopsy. They were fixed with 4% paraformaldehyde for 24 h, dehydrated, embedded in paraffin, and then sectioned. The paraffin slices were baked at 85 °C for 1 h on a slide rack, immersed in dewaxing solution, and then rehydrated using a gradient series of ethanol to water. Finally, HE and IHC staining were performed according to the following methods.

1. HE staining: Nuclei were stained with hematoxylin, differentiated by 1% hydrochloric acid alcohol, and turned blue with saturated lithium carbonate. Then, the slices were counterstained with eosin for 4 min. Finally, images were taken after dehydration, making the tissue transparent, and mounting
2. IHC: Dewaxed and hydrated slices were put into citrate solution for antigen retrieval and then incubated with 3% catalase solution at room temperature in the dark to remove endogenous hydrogen peroxide. The slices were blocked with 5% bovine serum albumin (BSA), incubated with primary antibody overnight at 4 °C, and then with secondary antibody at room temperature for 30 min. Next, they were stained with diaminobenzidine (DAB) and hematoxylin, differentiated by 1% hydrochloric acid alcohol, turned blue with saturated lithium carbonate, and then terminated with tap water. The final steps were the same as for HE staining. All reagents were from a universal two-step detection kit (PV-8000, Zhongshan Goldenbridge, Beijing, China)

### 2.4. Masson Staining

This experiment is conducted according with the instructions from the Masson staining kit (G1340, Solarbio Science & Technology, Beijing, China). The specific operation process is as follows: The above paraffin sections were mordanted with the mordant solution at room temperature overnight, and dyed with azurol blue staining solution; then, the Mayer hematoxylin staining solution was used for 2-3 min, respectively. Then, the sections were differentiated with acidic ethanol differentiation solution for several seconds until the tissue turned completely red. The sections were continuously stained with Ponceau fuchsin staining solution, differentiated with phosphomolybdic acid solution, and then stained with aniline blue. Finally, it was completed with dehydration, dewaxing, and sealing.

### 2.5. Western Blotting (WB)

Treated tissues or cells were washed with precooled PBS and then lysed with an appropriate volume of RIPA lysis buffer (P0013C, Beyotime, Haimen, China) containing protease inhibitors; following centrifugation, the supernatant was collected. The total protein concentration of each sample was determined by the bicinchoninic acid (BCA) method (Solarbio, Beijing, China). Protein samples were boiled in NuPAGE™ LDS sample buffer (4×) (#84788, Thermo Fisher Scientific, Waltham, MA, USA) for denaturation and stability. Then, 10% SDS-PAGE gels were prepared, and 40 *μ*g of protein samples were added. After the markers bands had completely separated by constant pressure electrophoresis, the proteins were transferred to a 0.22-*μ*m polyvinylidene difluoride (PVDF) membrane at constant pressure for approximately 90 min. Membranes were blocked with 5% skimmed milk at room temperature for 1 h, followed by incubation with primary and secondary antibodies, enhanced chemiluminescent (ECL) development, and imaging. Finally, the gray values of immunoreactive bands for each target protein were analyzed with Image J software. Statistical analyses were performed using Graph Prism 8 (GraphPad Software, San Diego, CA, USA). Information on all antibodies used for Western blotting are listed in Table 1.

### 2.6. Co-immunoprecipitation (Co-IP)

For these experiments, the crosslinked magnetic Co-IP Kit (#88805, Thermo Fisher Scientific, Waltham, MA, USA) was used according to its instructions. Specifically, protein A/G magnetic beads were pre-washed twice with 1× modified coupling buffer and then bound to the primary antibodies (SUMO1, SMAD4, or IgG). Then, the cells or tissues were collected and lysed with cooled IP lysis/wash buffer, and the lysate was incubated with antibody–magnetic beads overnight at 4 °C. Meanwhile, the input group was retained as a control. After the magnetic beads were washed with precooled IP lysis/wash buffer, the beads were collected, antigen was separated from the magnetic beads with elution buffer, and the supernatant was collected. After being neutralized with the neutralization solution, the eluant was boiled with 4× LDS sample buffer at 70 °C for 8 min. Next, target protein capture was detected according to the above WB experimental methods to determine the influence of each experimental factor on the protein-protein interaction.

### 2.7. Reactive Oxygen Species (ROS) Assay

Treated cells were digested with trypsin, collected, suspended in dichloro-dihydro-fluorescein diacetate (DCFH-DA) (10 *μ*M, diluted in serum-free medium), and then incubated for 20 min at 37 °C. The solution was mixed by inversion every 3 to 5 min to allow full contact between the probe and cells. Next, the cells were washed three times with serum-free medium to fully remove any DCFH-DA that had not entered cells. Finally, the fluorescence signals of cells in each group were directly observed and analyzed under confocal microscopy. For these assays, we used the Reactive Oxygen Species Assay Kit (S0033S, Beyotime).

### 2.8. Immunofluorescence (IF)

Treated A549 cells were fixed with 4% paraformaldehyde for 30 min and permeabilized with 0.3% Triton X-100 for 10 min at room temperature. After blocking with 1% BSA for 30 min at room temperature, the primary antibodies anti-SUMO1 (ab11672, Abcam, Cambridge, UK), anti-SMAD4 (#38454S, Cell Signaling Technology, Danvers, MA, USA), and anti-Vimentin (#5741S, Cell Signaling Technology) were diluted with PBS, and incubated overnight at 4 °C. Then, the cells were washed three times with PBS and incubated with the secondary antibodies goat anti-rabbit IgG H&L (Alexa Fluor^R^488) (ab150077, green) and goat anti-mouse IgG H&L(Alexa Fluor^R^594) (ab150116, red). Finally, an anti-fluorescence quenching sealing solution (including 4′,6-diamidino-2-phenylindole (DAPI)) was used to stain nuclei (blue) and seal the slides. Stained slides were observed under confocal microscopy. Finally, all steps after the inclusion of secondary antibody were performed in the dark.

### 2.9. Quantification of TGF-*β*1, TNF-*α*, and IL-6 in Mouse Lung Tissue (ELISA)

The contents of TGF-*β*1, TNF-*α*, and IL-6 were tested according to the instructions of the ELISA kit that all obtained from R&D Systems (Minneapolis, MN, USA).

### 2.10. Animals

All animal experiments performed in this study were approved by the Animal Medical Ethics Committee of the Fifth Central Hospital of Tianjin (TJWZX2021021). Briefly, 32 male C57BL/6 J mice (4-weeks-old, 18–20 g) were purchased from Sipeifu Biotechnology Co., Ltd (Beijing, China). The mice were placed in an environment with controlled temperature (22 °C–24 °C), stable humidity (40%–60%), and a 12-h light/dark cycle. The experiment was performed after 7 d of adaptive feeding.

SENP1 knockout (KO) animals were graciously provided by Professor Zhiqiang Liu (Tianjin Medical University, Tianjin, China) and Professor Yong Li (Shanghai Jiao Tong University School of Medicine, Shanghai, China). B6.129S-Sftpc tm1(cre/ERT2)Blh/J (#028054) mice were purchased from Jackson Laboratory (Bar Harbor, ME, USA). To create lung-specific SENP1 conditional KO (cKO) mice (SENP1^flox/flox^; Sftpc-cre), SENP1^flox/flox^ mice were crossed with Sftpc-cre mice. The SENP1^flox/flox^ pups produced in the same litter as the SENP1 cKO animals were included as littermate controls unless otherwise indicated.

### 2.11. Animal Protocol

Thirty-two male C57BL/6 J mice were randomly divided into four groups: control group, both infused and treated with normal saline only; BLM group, infused with BLM (5 mg/kg) and treated with normal saline; GA group, infused with saline and treated with GA (25 mg/kg/d); and BLM + GA group, infused with BLM (5 mg/kg) and treated with GA (25 mg/kg/d). After 1 week of adaptive feeding, mice in each group were anesthetized, slowly injected with the BLM solution (or saline control) into the trachea through the gap of the tracheal cartilage ring with a microsyringe, and quickly erected and rotated the mice's body to make the drugs evenly distributed in the lungs. Then, 24 h later, GA (or saline control) was administered after the BLM-induced PF model had been established. After treatment for 14 d, the mice were euthanized, and lung tissues were collected. The keft lungs were used for WBs and right lungs were fixed with 4% paraformaldehyde for HE, Masson, and IHC staining.

Similarly, SENP1 wild-type (WT) and KO mice derived in the C57BL/6 J strain were randomly divided into control, BLM, and BLM + GA groups. The modeling and treatment regimens were the same as above. Finally, the left lungs were used for WBs, while the right lungs were used for HE and Masson staining and ELISA.

### 2.12. Statistical Analysis

All experimental data are expressed as mean + standard error (SEM). Image J software was used to analyze the gray values of WB data, and GraphPad Prism 6 was used for data analysis and graphing. Statistical significance was determined using Student's *t*-test or one-way ANOVA followed by Tukey's test.

## 3. Results

### 3.1. Altered Expression of SUMO Family Members in Lung Tissues of IPF Patients

**Figure 1:**
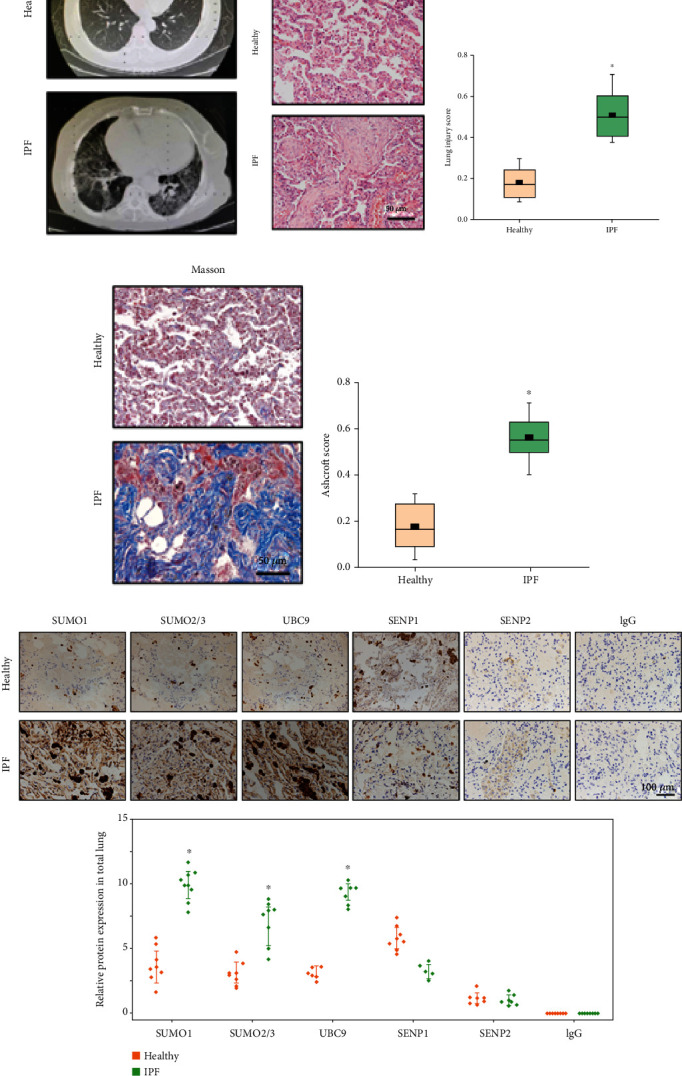
Differential expression of SUMO family members in lung tissues from idiopathic pulmonary fibrosis (IPF) patients and healthy controls. (a) Computed tomography (CT) images of lungs with cross sections of comparable anatomical locations. CT scans of IPF patients showed thickening of interstitial pulmonary lobules. (b, c) Representative HE and Masson's staining of lung tissues. Quantitative lung injury scores and Ashcroft scores are shown as box charts. (D) The expression of SUMO family members was examined by IHC, and their relative protein expression in total lung tissue is represented as a box chart. Data are presented as the mean ± SD. *n* = 6, ∗*P* < 0.05.

First, we tested the lung samples of healthy controls and IPF patients by longitudinal computed tomography (CT). The results showed that compared with healthy controls, the chest CTs of IPF patients presented a reticulated, cord-like, or patch-like density of increased shadow (Figure 1(a)). Next, HE and Masson staining of healthy and IPF tissue sections were performed. To further evaluate the lung injury in IPF samples, a pulmonary pathologist was consulted. The weighted comprehensive scoring method to evaluate neutrophil infiltration, signs of epithelial barrier defects, and structural damage in tissue sections was used. The results revealed a significant increase in structures associated with lung injury and inflammation in the IPF group compared with sections from healthy controls (Figure 1(b)). Consistently, Masson staining revealed significantly increased levels of overall fibrosis and structural lung injury in the IPF group (Figure 1(c)).

Recent studies have shown that SUMOylation is involved in the pathogenesis of fibrosis [35], especially myocardial fibrosis [36]. Thus, it remains unclear whether IPF is related to SUMOylation. Here, we used IHC to determine that the protein levels of SUMO1, SUMO2/3, and UBC9 were significantly increased in the whole lung tissue of IPF patients compared with those of healthy lung tissue. Meanwhile, SENP1 protein expression was downregulated, and there was no significant change in SENP2. The changes in the expression of these proteins were obvious, especially in epithelial cells of IPF patients (Figure 1(d)). These preliminarily results suggested that PF is related to key proteins in the SUMO pathway; thus, we focused on the changes in SUMO1 and SENP1 in subsequent experiments.

### 3.2. GA Regulated SUMO1, UBC9, and SENP1 Expression in a BLM-Induced Mouse Model of PF

**Figure 2:**
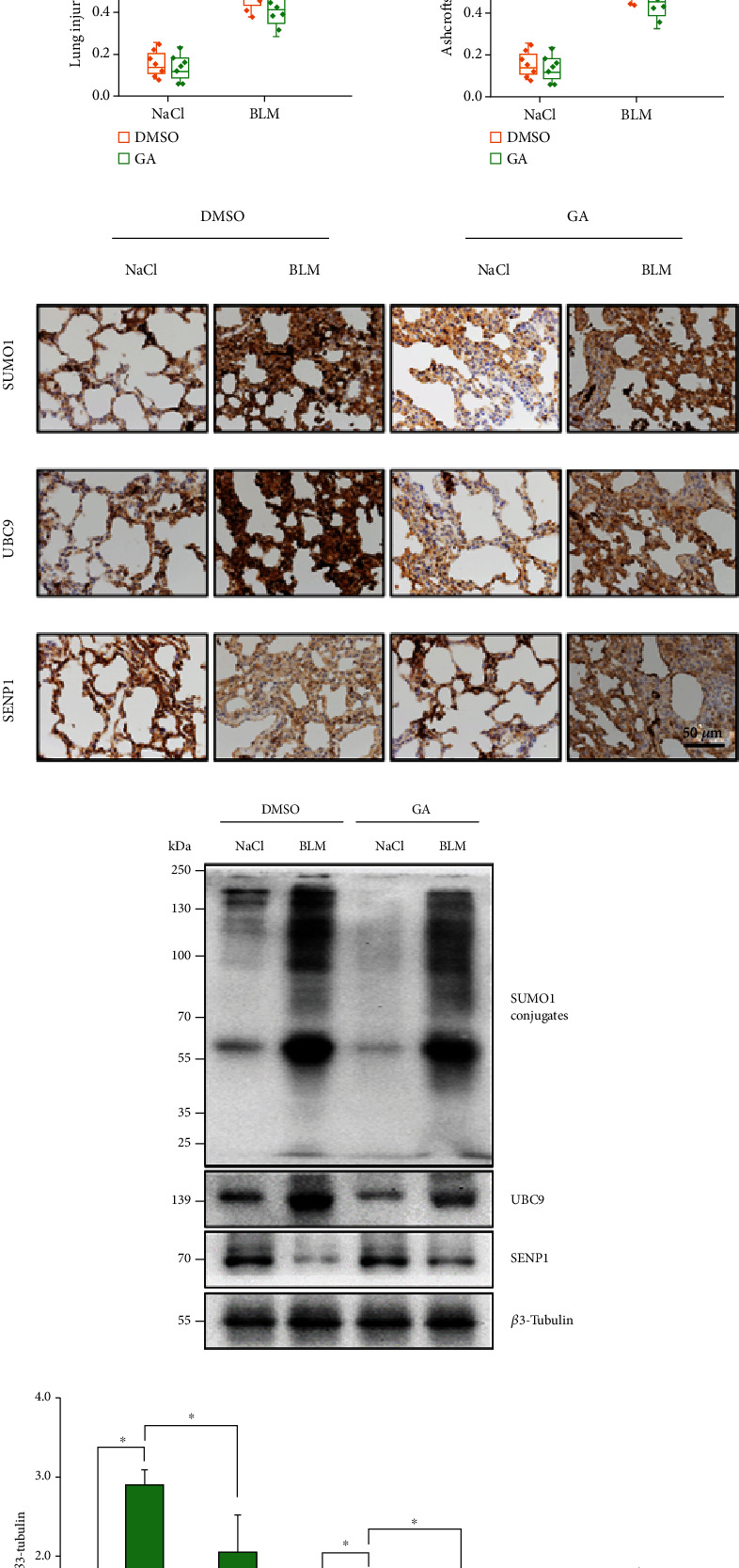
Ginkgolic acid (GA) regulated the expression of key proteins in the SUMOylation pathway *in vivo*. Mice were challenged with bleomycin (BLM) through the airway, and GA was administered through intraperitoneal injection 24 h later. Meanwhile, the control group was administered and treated with normal saline (*n* = 8). (a) Images of representative HE and Masson staining of lung tissues from mice in the indicated groups. Quantitative lung injury scores and Ashcroft scores are shown as box charts. Scale bar: 100 *μ*m. (b) SUMO1, UBC9, and SENP1 protein levels were detected by IHC and WB. Data are presented as the mean ± SD. *n* = 6, ∗*P* < 0.05.

Studies have shown that GA can inhibit protein SUMOylation [37]. For this reason, we hypothesized that GA might reduce the occurrence and development of PF through the SUMOylation of regulatory proteins. To test this hypothesis, we constructed a mouse model of PF and obtained lung tissue after GA administration. Then, the effects of GA on PF were detected by HE and Masson staining (Figure 2(a)). HE results showed that structures associated with lung damage and inflammation were significantly increased in the BLM model group compared with those in the control group, and this effect could be blocked by GA treatment. Masson staining results also showed that compared with those in control group, there were significantly increased levels of overall fibrosis and structural lung injury in the BLM model group, and this effect of BLM could be inhibited by GA treatment. These results showed that GA could reduce the occurrence of BLM-induced PF. Next, we further explored the relationship between GA treatment of murine PF and protein SUMOylation by IHC and WB. These results showed that the SUMO1 modification of proteins and the expression of UBC9 were increased in the BLM model group, while SENP1 protein expression was decreased in the BLM group compared with that in the controls by IHC and WB; all of these changes were inhibited by GA treatment (Figures 2(b)–2(c)). This indicated that GA could reverse the increased SUMOylation levels and the changes in Ubc9 and SENP1 protein expression induced by BLM.

### 3.3. GA Prevented PF by Elevating SENP1 Expression *In Vivo*

**Figure 3:**
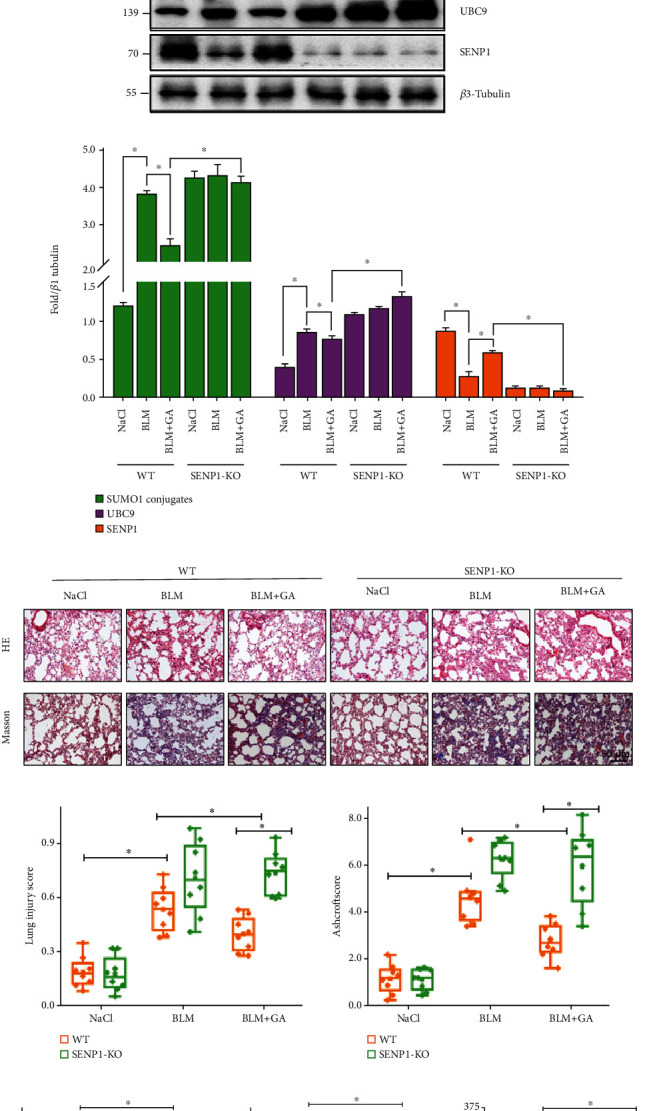
Ginkgolic acid (GA) prevented pulmonary fibrosis by increasing SENP1 expression and activity *in vivo*. Wild-type (WT) mice and SENP1-knock out (KO) transgenic mice were challenged with bleomycin (BLM) through the airway and treated by intraperitoneal administration of GA after 24 h. Meanwhile, the control group was administered and treated with normal saline (*n* =8). (a) SUMO1, UBC9, and SENP1 protein levels in lung tissues from the different groups were detected by WB. (b) Images of representative HE and Masson staining of lung tissues from the indicated groups. Quantitative lung injury scores and Ashcroft scores are shown as box charts. Scale bar: 100 *μ*m. (c) ELISA kits were used to detect levels of TGF-*β*1, TNF-*α*, and IL-6 in lung tissues of each group. Data are presented as the mean ± SD. *n* = 6, ∗*P* < 0.05.

To determine whether elevated SENP1 levels influenced the protective effects of GA, SENP1-KO mice were subjected to the murine PF model of topical BLM administration. As shown in Figures 3(a)–3(b), we observed decreased SENP1 protein levels and variable increases in SUMO1 conjugation and UBC9 expression in SENP1-KO mice, indicating successful construction of SENP1-KO transgenic mice. Subsequently, we detected the effects of SENP1-KO on lung fibrosis by HE and Masson staining following BLM administration (Figures 3(c)–3(d)). HE results showed that compared with the BLM model group, the GA administration group had significantly reduced structural lung damage and inflammation, which was inhibited in SENP1-KO animals. Similarly, Masson staining results showed that overall levels of fibrosis and structural lung injury were significantly improved following GA administration, but this effect was lost in SENP1-KO mice. These results indicated that GA prevented PF through SENP1. TGF-*β*1 is widely recognized as a key fibrogenic cytokine [38]. We used ELISA to detect changes in TGF-*β*1 and the inflammation-related factors TNF-*α* and IL-6 in lung tissues from the various group. Levels of TGF-*β*1, TNF-*α*, and IL-6 were decreased in lung tissues from the GA group, and this effect was abolished in the SENP1-KO group (Figure 3(e)).

### 3.4. GA Mediated TGF-*β*1-Induced SMAD4 SUMOylation

**Figure 4:**
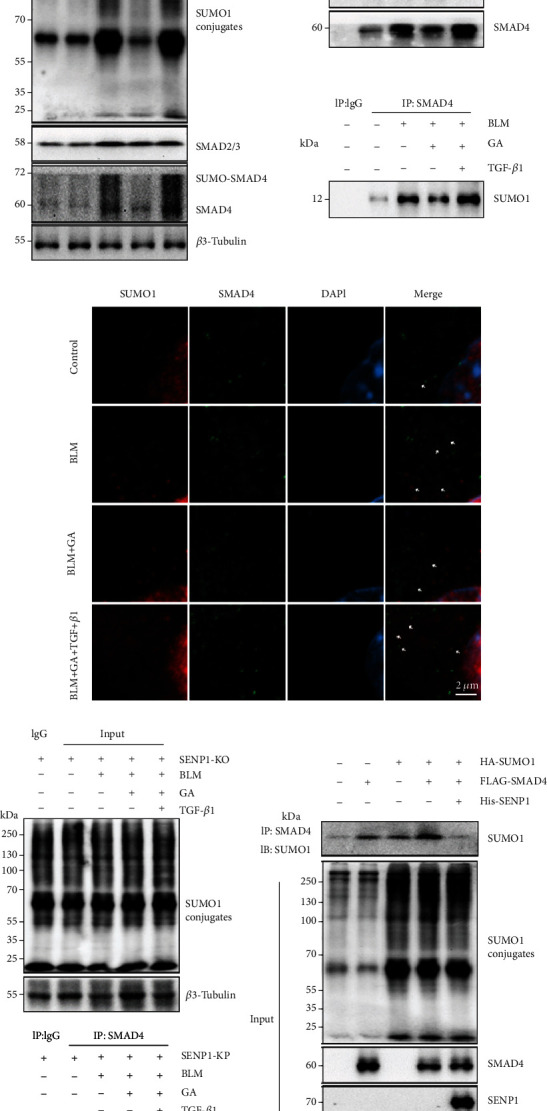
Ginkgolic acid (GA) mediated TGF-*β*1-induced SMAD4 SUMOylation in a mouse model of pulmonary fibrosis. (a) Lung tissues of wild-type (WT) mice in the different administration/treatment groups were used in Co-IP experiments with anti-SUMO1and anti-SMAD4 antibodies, followed by western blot analysis with the indicated antibodies (input). The results showed that TGF could reverse the inhibitory effect of GA on the interaction between SUMO1 and SMAD4, not SMAD2/3. (b) The effect of GA on SMAD4 SUMOylation in WT mice was analyzed by immunofluorescence. (c) Lung tissues of SENP1-KO transgenic mice in the different administration/treatment groups were used in Co-IP experiments as in (a). (d) The effect of SENP1 on the SUMOylation of SMAD4 was investigated by Co-IP in A549 cells transfected with the indicated plasmids.

Studies have shown that GA can regulate TGF-*β*1-induced SMAD4 SUMOylation [39]. So, we next investigated whether the ability of GA to inhibit lung fibrogenesis depended on this activity. Total protein from the lungs of treated WT mice was extracted. WB results showed that compared with that in the BLM model group, there was reduced protein expression of SUMO1 conjugates and SMAD2/3/4 in the GA group; TGF-*β*1 could conversely inhibit the effect of GA on the expression of SUMO1 and SMADs. Besides, not SMAD2/3, only SMAD4 showed altered expression change of SUMO-modified bands, which was also confirmed by the Co-IP results. GA inhibited the interaction, but this could be reversed by TGF-*β*1 (Figure 4(a)). Additionally, confocal microscopy showed that compared with the BLM group, GA reduced the co-localization of SUMO1 and SMAD4; this effect could also be reversed by TGF-*β*1 (Figure 4(b)). These data indicated that GA may participate in lung fibrogenesis by regulating the TGF-*β*1-induced SUMOylation of SMAD4.

To verify the role of SENP1 in the GA-mediated regulation of TGF-*β*1-induced SMAD4 SUMOylation, we conducted Co-IP experiments using lung tissues from SENP1-KO mice. As shown in Figures 4(a) and 4(c), we found that compared with the BLM + GA group, TGF-*β*1 promoted the interaction between SUMO1 and SMAD4, but this was blocked in SENP1-KO mice. To further verify the role of SENP1 in the SUMO1 modification of SMAD4, we transfected HA-SUMO1, FLAG-SMAD4, and His-SENP1 plasmids into A549 cells. The results showed that SMAD4 SUMOylation was only increased when HA-SUMO1 and FLAG-SMAD4 plasmids were co-transfected. However, when HA-SUMO1, FLAG-SMAD4, and His-SENP1 were co-transfected, the SUMO1 modification of SMAD4 was decreased (Figure 4(d)). These indicated that SENP1 can block the covalent binding of SMAD4 to SUMO1.

### 3.5. SENP1 Mediated the DeSUMOylation of SMAD4 in Response to TGF-*β*1-Induced EMT

**Figure 5:**
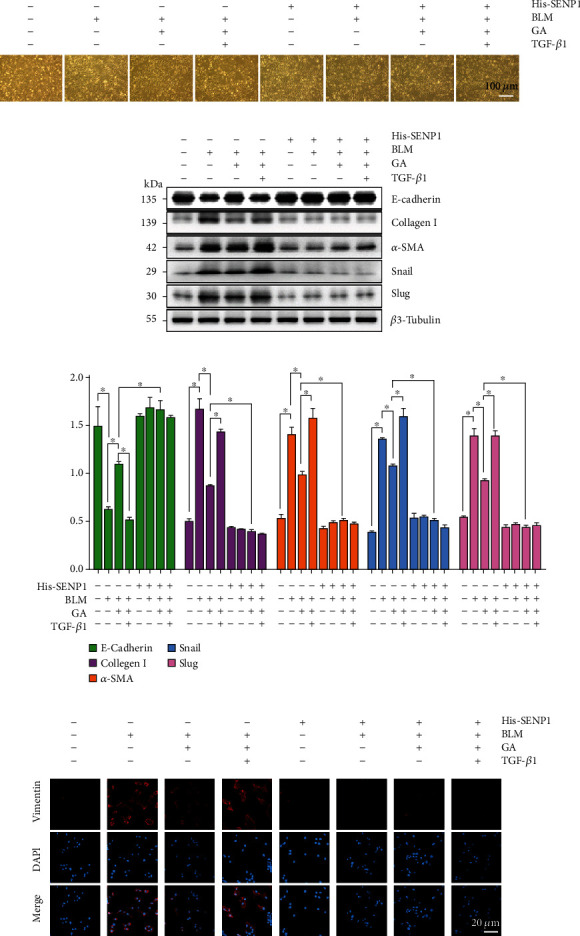
SENP1 mediated the deSUMOylation of SMAD4 in response to TGF-*β*1-induced EMT. A549 cells were either transfected with His-SENP1 or treated with BLM, GA, NAC (TGF-*β*1 scavenger agonist), or them in combination. (a) The morphological changes of cells in each group were observed under light microscopy. (b) Western blot (WB) analysis of the levels of key EMT-related proteins. (c) Vimentin expression was detected by immunofluorescence to determine the degree of pulmonary fibrosis. Data are presented as the mean ± SD. *n* = 6, ∗*P* < 0.05.

Next, we further tested whether SENP1 regulation of SMAD4 SUMOylation was involved in TGF-*β*1-induced EMT. Observations of cell morphology revealed that compared with the BLM group, GA inhibited the transformation of epithelial-like cells to fibroblast-like cells, which was reversed by TGF-*β*1; meanwhile, His-SENP1 significantly inhibited the transformation of epithelial cells to a fibroblast-like morphology (Figure 5(a)). WB results also showed that compared with that in the BLM group, E-cadherin protein expression was significantly increased in the GA group, while collagen I and *α*-SMA were significantly decreased. These effects were reversed by TGF-*β*1, while His-SENP1 significantly blocked the activity of TGF-*β*1 (Figures 5(b)–5(c)). The transcription factors Snail and Slug are the dominant transcriptional repressors of E-cadherin [40], and we subsequently tested whether GA mediated their expression. As shown in Figures 5(b)–5(c), Snail and Slug expression were significantly decreased in the GA group compared with those in the BLM group, and this effect was reversed by TGF-*β*1; His-SENP1 significantly blocked the effects of TGF-*β*1. Similarly, IF results showed significantly decreased vimentin expression in the GA group compared with that in the BLM group, and this effect was reversed by TGF-*β*1, while His-SENP1 could significantly inhibit this activity of TGF-*β*1 (Figure 5(d)). In summary, SMAD4 SUMOylation was regulated by SENP1 and was involved TGF-*β*1-induced EMT.

### 3.6. SENP1 Mediated DeSUMOylation of SMAD4 in Response to TGF-*β*1-Induced ROS Production

**Figure 6:**
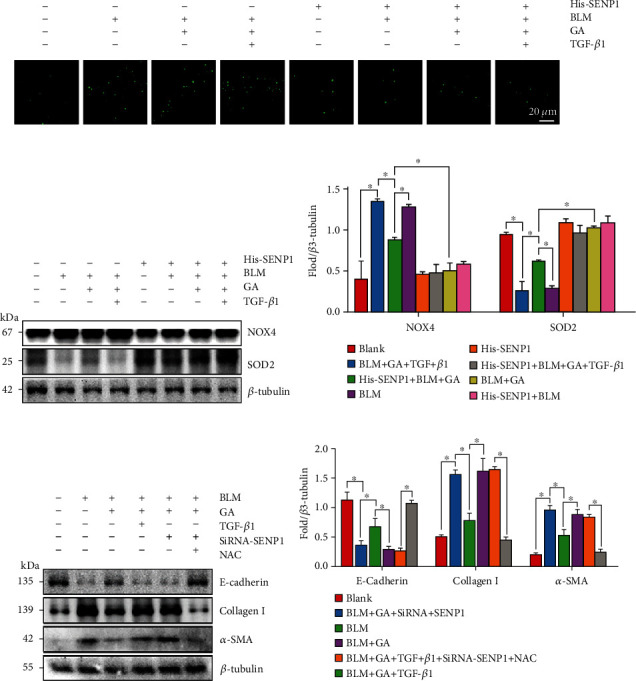
SENP1 mediated the deSUMOylation of SMAD4 in response to TGF-*β*1-induced ROS generation. A549 cells were either transfected with His-SENP1 or treated with BLM, GA, TGF-*β*1, or them in combination. (a) The fluorescence intensity of 2′,7′-dichlorofluorescein (DCF) in cells of each group was detected using a reactive oxygen species (ROS) assay kit to show ROS levels in cells of each treatment group. (b) WB analysis of the key oxidases that triggered the ROS outbreak to clarify the role of SMAD4 SUMOylation and SENP1 in TGF-*β*1-induced ROS generation. (c) A549 cells transfected with siRNA-SENP1 or treated with BLM, GA, TGF-*β*1, NAC, or them in combination were subjected to WB analysis to detect the levels of key protein involved in EMT. Data are presented as the mean ± SD. *n* = 6, ∗*P* < 0.05.

Previous studies have confirmed that ROS mediates a variety of cellular functions including EMT [41]; thus, we further investigated whether SENP1-regulated SMAD4 SUMOylation affected ROS production during TGF-*β*1-induced EMT. Confocal microscopy results showed that compared with that in the BLM group, there was significantly decreased 2′,7′-dichlorofluorescein (DCF) fluorescence intensity in the GA group. However, this effect was reversed by TGF-*β*1, while His-SENP1 significantly inhibited TGF-*β*1 activity (Figure 6(a)).

An imbalance between oxidative and antioxidant enzymes can cause abnormal ROS production [42]. For example, increased NADPH oxidase 4 (NOX4) activity and decreased SOD2 expression can cause a ROS outbreak [43]. We performed WBs to detect changes in the expression of these proteins in each treatment group. As shown in Figures 6(b)–6(c), NOX4 expression was decreased in the GA group, while SOD2 protein expression increased, which were compared with that in the BLM group. This effect was reversed by TGF-*β*1, and His-SENP1 significantly inhibited the activity of TGF-*β*1. These results indicated that SENP1-regulated SMAD4 SUMOylation participates in antioxidant effects downstream of TGF-*β*1-induced EMT. We further verified these results with the ROS scavenger N-Acetyl-L-cysteine (NAC). WB results showed that compared with the BLM group, E-cadherin protein expression was significantly increased in the GA group, while collagen I and *α*-SMA showed decreased expression. This effect was reversed by TGF-*β*1 and siRNA-SENP1, while NAC significantly improved the effects of TGF-*β*1 and siRNA-SENP1 (Figures 6(d)–6(e)). These results indicated that GA could prevent the oxidative stress induced by TGF-*β*1 through SENP1 and closely related to EMT.

## 4. Discussion

IPF is a progressive chronic PF disease [44]. The etiology of IPF is complex, its pathogenesis has not been fully elucidated, and disease progression and survival prognoses are heterogeneous. This study revealed the following points: (1) GA could protect against BLM-induced PF; (2) GA could reduce PF induced by the increased SUMOylation of SMAD4 through SENP1; (3) SMAD4 SUMOylation participated in TGF-*β*1-mediated lung EMT, which was accompanied by increased ROS production.

GA is an important chemical component of *G. biloba* extract [8]. Previous studies have reported that *G. biloba* extract can prevent renal fibrosis by modulating Akt/mTOR signaling in diabetic nephropathy [45]; it can also reduce the occurrence of liver fibrosis through NF-*κ*B and TGF-*β*1 or by regulating p38 MAPK, NF-*κ*B, and Bcl-2/Bax signaling to relieve liver fibrosis [46]. GA is small-molecule compound extracted from Ginkgo biloba. At present, the reports showed that GA could pass through the blood-brain barrier orally and its bioavailability is also good [8, 47]. And studies reported that it has various biologically relevant activities, including antitumor properties, and recent studies showed that it also had a protective effect on myocardial fibrosis caused by myocardial infarction through the SUMOylation of TGF-*β*1 [13]. On the basis of these data, we hypothesized that GA might have a protective effect against PF and that its mechanism of action would be related to TGF-*β*1 signaling and protein SUMOylation, which was verified in the BLM-induced mouse model of PF.

The covalent binding of SUMO to target proteins is a post-translational modification in eukaryotic cells that makes proteins more stable and thus regulates many key cellular activities, such as gene expression, nuclear transport, signal transduction, cell cycle progression, and apoptosis [48, 49]. Additionally, SUMOylation can also participate in anti-inflammatory and fibrotic processes through peroxisome proliferator-activated receptor-*γ* (PPAR-*γ*)-, NF-*κ*B-, and TGF-*β*-dependent pathways [35, 50]. Current studies have found that SUMOylation is involved in fibrogenesis of multiple organs, such as renal fibrosis, liver fibrosis, and myocardial fibrosis [13, 51, 52]. Among them, there are reports that PIAS4 (the E3 ligase of the SUMOylation pathway) can regulate fibrotic lung injury through the TGF-*β* pathway [53], indicating that SUMOylation plays a role in PF. In this study, we confirmed the role of SUMOylation in PF and showed that the protective effect of GA against PF was related to this mechanism.

TGF-*β*1 is a fibrogenic factor, and the SMAD family comprises signal transduction molecules in the TGF-*β*1 pathway [27]. It is currently believed that extracellular matrix deposition and excessive fibrosis are caused by increased TGF-*β*/SMAD signaling [54]. Studies have found that the SUMOylation of SMAD4 induced by SUMO2/3 plays a crucial role in renal fibrosis associated with diabetic nephropathy [34]. GA can alleviate myocardial infarction-induced cardiac dysfunction and fibrosis through SUMOylation of the PML/Pin1/TGF-*β*1 pathway [13]. On the basis of these findings, we newly discovered the role of SUMOylated SMAD4 in the improvement of PF by GA and that knocking out SENP1 could inhibit the effect of GA on PF.

Increasing evidence has shown that activation of TGF-*β*1/SMAD signaling is the primary driving force of fibrosis [27] and oxidative stress is an important harmful promoting factor related to the fibrogenic activity of TGF-*β*1/SMAD [55]. There is a significant correlation between TGF-*β*1 and oxidative stress signals throughout the process of fibrosis, and TGF-*β*1 can increase ROS levels in fibrotic pulmonary tissue [56]. Additionally, SMAD4 is a SUMO-modified protein [57]. Herein, we showed that SMAD4 played an important role in the improvement of PF by GA. Thus, we next investigated whether it affected ROS production. Nicotinamide adenine dinucleotide phosphate (NADPH) oxidase is the main source of endogenous ROS [58]. NOX4 has a unique compositional activity. As the primary enzymatic source of extracellular ROS, NOX4-derived ROS has been recognized as the main source of oxidative stress [59]. NOX4 acts as a downstream mediator of the profibrotic responses mediated by TGF-*β*1 and induces ROS production, which plays an important role in the pathogenesis of liver, kidney, lung, and heart fibrosis. NOX4 is a potential therapeutic target related to PF and other diseases associated with enhanced TGF-*β*1 signaling [60]. SOD is a classical antioxidant enzyme, and its expression indirectly reflects the oxidation state of lung tissue [61]. Therefore, we then tested these two indicators and found that GA inhibited the burst of ROS by regulating the SUMOylation of SMAD4, thereby alleviating PF.

EMT is a key step in the pathogenesis of PF [62]. TGF-*β*1 signaling is generally recognized to mediate and regulate EMT [63]. Thus, we tested the expression of key proteins in the EMT pathway, which further confirmed that GA reduced EMT and improved the pathogenesis of PF by regulating SMAD4 SUMOylation.

## 5. Conclusions

In summary, we found that GA regulates the TGF-*β*1-mediated SUMOylation of SMAD4 through SENP1, inhibiting bursts of ROS, which reduces the occurrence of EMT, ultimately achieving protective effects against PF. However, the exact molecular mechanism underlying this activity is still not perfectly understood, and therefore further research and *in vivo* verification are needed.

## Figures and Tables

**Table 1:** Information on all antibodies used in this study.

Antibody	Company	Code	Application	Dilution
SUMO1	Abcam	ab11672	WB	1 : 1000
UBC9	Abcam	ab75854	WB	1 : 2000
SENP1	Abcam	ab108981	WB	1 : 4000
*β*3-tubulin	CST	#5666	WB	1 : 2000
SMAD2/3	Abcam	ab202445	WB	1 : 1000
SMAD4	Abcam	ab230815	WB	1 : 1000
E-cadherin	BD	610404	WB	1 : 500
Collagen I	Abcam	ab270993	WB	1 : 1000
*α*-SMA	CST	#19245	WB	1 : 1000
Snail	CST	#3879	WB	1 : 1000
Slug	CST	#9585	WB	1 : 1000
NOX4	Abcam	ab133303	WB	1 : 2000
SOD2	CST	#13141	WB	1 : 1000

## Data Availability

All the raw data in this article are obtained by the authors through experiments, without any reservations, and have not been reused in other articles.
